# APSP Journal of Case Reports: A New Beginning

**Published:** 2010-08-14

**Authors:** Bilal Mirza, Jamshed Akhtar

**Affiliations:** Department of Paediatric Surgery, The Children's Hospital and The Institute of Child Health Lahore, Pakistan; Department of Pediatric Surgery, National Institute of Child Health Karachi, Pakistan

In a book, Orphan Diseases: New Hope for Rare Medical Conditions, author Wendy Murphy in introductory pages quotes William Harvey, an English physician (1578 - 1657) ”Nature is nowhere accustomed more openly to display her secret mysteries than in cases where she shows tracings of her workings apart from the beaten paths; nor is there any better way to advance the proper practice of medicine than to give our minds to the discovery of the usual law of nature, by careful investigation of cases of rarer forms of disease” [1 , 2].

This is apt to the discipline of pediatric surgery, as unusual anomalies are seen frequently in clinical practices. At times one tries to seek opinions and learn from experiences of other colleagues in dealing with such patients. Case reports thus become an important source of documenting such experiences and observations. In hierarchy of evidence in scientific literature, they are placed at lowest level but even then, are the important contribution to the medical literature. According to Karl Popper any proposition or theory is scientific only if it is falsifiable. Thus logically an observation can falsify an assertion. A reported case may shift the existing thinking paradigm [3].

Case reports have made important contributions in relation to many clinical conditions. Cases of HIV / AIDS have been documented initially as case reports. Many adverse drug reactions came into light when such incidences were reported under this category of manuscript. They are of educational value and guide us to future areas of research. In selecting case reports for this journal we ensure that they must address or reveal new scientific information. We have also added segments like images and letters to the editor, so as to provide educationally rich clinical observations to our readers.

Many scientific journals have stopped publication of case reports. Various reasons have been cited though according to some publishing of case reports affects the impact factor [4]. Also as research became an integral part of teaching, training and promotions, authors are now inclined towards more elaborate study designs, thus their interest in this category of publication has declined. This domain is usually left to the juniors in the discipline. The APSP Journal of Case Reports, an online, free access periodical, from the platform of the Association of Paediatric Surgeons of Pakistan, is one such portal through which authors can present their experiences to the world.

The first issue of this journal is taken out in record time. One of the editors (Bilal Mirza) remained instrumental in this regard. He contributed greatly in developing and maintaining the website of the journal as well. All articles presented in this are peer reviewed by senior pediatric surgeons both from Pakistan and abroad. Elaborate guidelines are available on our website both for the authors and reviewers. We hope you will contribute to this first ever electronic journal dedicated to case reports from Pakistan. Your participation by submitting quality manuscript shall improve the standard of the journal and increase its readership.

## Footnotes

**Source of Support:** Nil

**Conflict of Interest:** None declared
